# Supplementary material for: Safety Profile of Medicines Used for the Treatment of Drug-Resistant Tuberculosis: A Descriptive Study Based on the WHO Database (VigiBase®)
Source: Antibiotics (Basel). 2023 Apr 25;12(5):811. doi: 10.3390/antibiotics12050811 (PMC10215388; doi:10.3390/antibiotics12050811)
Supplement: Supplementary file 1 [file antibiotics-12-00811-s001.zip › antibiotics-2306343-supplementary.pdf]

Supplementary Data

Table S1: A cross tabulation analysis of the most frequent ADRs associated with top 5 withdrawn medicines

			The most frequent ADRs associated with top 5 withdrawn medicines						Total
			Ear and labyrinth disorders	Gastrointestinal disorders	Nervous system disorders	Psychiatric disorders	Skin and subcutaneous tissue disorders	Others	
Adolescent	Most frequent withdrawn medicines	Cycloserine	0	0	3	9	1	17	30
			0.0%	0.0%	10.0%	30.0%	3.3%	56.7%	100.0%
		Linezolid	0	1	7	0	0	27	35
			0.0%	2.9%	20.0%	0.0%	0.0%	77.1%	100.0%
		Pyrazinamide	0	2	3	0	4	16	25
			0.0%	8.0%	12.0%	0.0%	16.0%	64.0%	100.0%
		Kanamycin	17	0	1	0	1	4	23
			73.9%	0.0%	4.3%	0.0%	4.3%	17.4%	100.0%
		Ethionamide	0	1	2	1	4	13	21
			0.0%	4.8%	9.5%	4.8%	19.0%	61.9%	100.0%
		Others	1	15	6	4	9	112	147
			0.7%	10.2%	4.1%	2.7%	6.1%	76.2%	100.0%
	Total		18	19	22	14	19	189	281
			6.4%	6.8%	7.8%	5.0%	6.8%	67.3%	100.0%
Adult	Most frequent withdrawn	Cycloserine	4	19	31	168	8	30	260
			1.5%	7.3%	11.9%	64.6%	3.1%	11.5%	100.0%
		Linezolid	0	19	52	2	10	112	195
			0.0%	9.7%	26.7%	1.0%	5.1%	57.4%	100.0%
		Pyrazinamide	4	32	5	7	18	127	193

			2.1%	16.6%	2.6%	3.6%	9.3%	65.8%	100.0%
		Kanamycin	150	28	12	2	17	53	262
			57.3%	10.7%	4.6%	0.8%	6.5%	20.2%	100.0%
		Ethionamide	4	89	13	13	17	50	186
			2.2%	47.8%	7.0%	7.0%	9.1%	26.9%	100.0%
		Others	29	139	54	29	72	497	820
			3.5%	17.0%	6.6%	3.5%	8.8%	60.6%	100.0%
	Total		191	326	167	221	142	869	1916
			10.0%	17.0%	8.7%	11.5%	7.4%	45.4%	100.0%
Elderly	Most frequent withdrawn medicines	Cycloserine	0	0	0	4	0	2	6
			0.0%	0.0%	0.0%	66.7%	0.0%	33.3%	100.0%
		Linezolid	0	0	0	0	0	2	2
			0.0%	0.0%	0.0%	0.0%	0.0%	100.0%	100.0%
		Pyrazinamide	2	2	3	1	1	13	22
			9.1%	9.1%	13.6%	4.5%	4.5%	59.1%	100.0%
		Kanamycin	18	3	3	0	0	6	30
			60.0%	10.0%	10.0%	0.0%	0.0%	20.0%	100.0%
		Ethionamide	2	3	2	1	0	5	13
			15.4%	23.1%	15.4%	7.7%	0.0%	38.5%	100.0%
		Others	6	0	7	0	0	51	64
			9.4%	0.0%	10.9%	0.0%	0.0%	79.7%	100.0%
	Total		28	8	15	6	1	79	137
			20.4%	5.8%	10.9%	4.4%	0.7%	57.7%	100.0%
Total	Most frequent withdrawn	Cycloserine	4	19	34	181	9	49	296
			1.4%	6.4%	11.5%	61.1%	3.0%	16.6%	100.0%
		Linezolid	0	20	59	2	10	141	232
			0.0%	8.6%	25.4%	0.9%	4.3%	60.8%	100.0%
		Pyrazinamide	6	36	11	8	23	156	240

		2.5%	15.0%	4.6%	3.3%	9.6%	65.0%	100.0%
	Kanamycin	185	31	16	2	18	63	315
		58.7%	9.8%	5.1%	0.6%	5.7%	20.0%	100.0%
	Ethionamide	6	93	17	15	21	68	220
		2.7%	42.3%	7.7%	6.8%	9.5%	30.9%	100.0%
	Others	36	154	67	33	81	660	1031
		3.5%	14.9%	6.5%	3.2%	7.9%	64.0%	100.0%
Total		237	353	204	241	162	1137	2334
		**10.2%**	**15.1%**	**8.7%**	**10.3%**	**6.9%**	**48.7%**	**100.0%**
